# Cholera crisis persists: A call for integrated health strategies

**DOI:** 10.1371/journal.pntd.0013272

**Published:** 2025-07-23

**Authors:** Musawer Khan, Kamil Ahmad Kamil

**Affiliations:** 1 Combined Military Hospital Quetta, Quetta, Pakistan; 2 Internal Medicine Department, Mirwais Regional Hospital, Kandahar, Afghanistan; Menzies School of Health Research, AUSTRALIA

## Dear Editor,

Despite the implementation of global health strategies, cholera deaths continue to rise, highlighting the sobering realization that vaccines alone cannot stem the tide without robust infrastructure. Current approaches require critical reevaluation in light of a 71% increase in cholera mortality in 2023 and a 54% increase in deaths despite declining cases in 2024 [1]. This letter highlights systemic barriers such as persistent conflicts, climate-induced floods, and chronic sanitation deficiencies while advocating for a transformative shift toward equitable, evidence-based interventions.

Data from a significant 2023 interventional trial conducted in Afghanistan, the Democratic Republic of the Congo, Malawi, Mozambique, and Haiti shows that a single-dose oral cholera vaccine (OCV) led to a 38% drop in cholera incidence and a 45% decrease in severe diarrhea among 12,500 vaccinated individuals compared to controls [2]. However, the minimal 0.4% difference in mortality (0.8% vaccinated versus 1.2% control) emphasizes the limitations of vaccination in addressing late-stage illness, especially where healthcare resources are severely constrained. This aligns with UN data from 2024, suggesting that healthcare system failures, rather than prevention shortcomings, are driving mortality [1].

Preliminary WHO forecasts for 2025 predict a 15% rise in cholera outbreaks linked to intensified flooding events [3]. Mozambique’s refugee camps, for instance, reported a 60% higher death rate in 2024 due to inadequate healthcare access. The WHO’s unfulfilled $50 million funding appeal for 2024 further highlights critical gaps needing urgent attention.

Integrated Water, Sanitation, and Hygiene (WASH) interventions offer strong evidence for reducing cholera incidence. A 2023 prospective cohort study from Kolkata, India revealed a 30% lower risk of cholera in households practicing improved WASH measures over five years, with children under five experiencing the most significant benefits [4]. Practices such as regular handwashing, access to safe water, and the use of private or communal flush toilets were key contributors.

However, implementation remains slow, particularly in conflict zones where disrupted vaccine cold chains may further compromise intervention effectiveness—an area requiring further investigation. Future research should explore longitudinal data on the efficacy of multi-dose OCV in displaced populations to identify critical thresholds for infrastructural interventions.

To effectively reduce cholera-related mortality, policymakers must immediately allocate 30% of cholera response budgets to WASH infrastructure by 2026, with measurable targets for clean water access in endemic areas. Short-term strategies focusing solely on vaccination are insufficient. A comprehensive, equity-driven approach integrating WASH initiatives with immunization efforts is essential to combat the cholera crisis.

Stakeholders, including researchers and legislators, must champion feasible, integrated solutions to alter the trajectory of the ongoing pandemic.
